# Notes on the Breeding of a Hippopotamus

**Published:** 1890-01

**Authors:** W. A. Conklin

**Affiliations:** Director New York Zoological Gardens


### NOTES ON THE BREEDING OF A HIPPOPOTAMUS.

By W. A. Conklin, PhD., D.V.S.,
*Director New York Zoological Gardens.*

On Sunday December 1, 1889, at about noon, I noticed that the female hippopotamus became very uneasy and gave indications of calving. I gave orders to have the house cleared of visitors, also to separate the male from the female. For the previous two weeks the female had been leading her companion a rather disagreeable life by driving him away from her presence whenever he came near her, showing that she desired to be left alone. At four o'clock in the afternoon the membranes protruded and eight hours later the infant hippopotamus was born, coming head first. The placenta which came away shortly after was sent to Dr. A. Liautard, Am. Vet. College, who reports it equine in appearance. In about three hours the young animal walked about its cage and around its mother who appeared to be very fond of it and followed it about. Soon after the young one selected a spot in the cage where it remained with the mother by its side. The following day it left its bed several times, making three or four turns about the cage and then returning. At these times the mother always kept near it and acted as if she wanted it to nurse. Tuesday morning, thirty-six hours after birth, I found it was growing weaker, and as it had not nursed I decided to remove it and feed it by hand. After several attempts it readily sucked from a rubber tube fitted to a bottle containing diluted cow's milk, drinking from one to two quarts at intervals of four hours. On Thursday afternoon I noticed a listlessness in the animal, with a high fever, also a disinclination for food. Believing it to be a cold caused by a fall of temperature and draught on Tuesday night I ordered mustard baths and gave quinine, but it remained in the same listless state until 4 o'clock Friday morning when death took place. Post-mortem revealed congestion of the right lung. On the second day I noticed on the baby the bloody sweat peculiar to the old animals. On the fourth day an opacity of the right cornea became apparent. Dr. Spitzka writes me as follows :—

"In the winter of 1877–78, there died of pneumonia, a hippotamus between two and three years old, belonging to P. T. Barnum. I obtained the brain of this animal, and published the post-mortem findings in *Der Zoologischer Garten*, 1880–81. During the life of the animal, I frequently observed the well-known phenomenon of "bloody perspiration," and the periodical corneal opacity to which I referred when we examined the body of the baby hippopotamus a few days ago. Mr. Kahn, then in charge of the former animal, first directed attention to the fact about which both Dr. Dorner and myself were skeptical. However, Mr. Kahn convinced both of us, that every twenty-eight days or thereabouts, first one cornea, and then the other became opaque. The phenomenon lasted fully three days, and was unsymmetrical, beginning earlier in one eye than in the other, the former having cleared up, as the latter passed through the process. It was more severe in one eye, at least on the *four* occasions when I studied it. The opacity distinctly disturbed vision, the animal acting stupid-like but being constitutionally only slightly affected. At the height of the process, the entire cornea appeared bluish, and the iris could not be distinguished through it. There was no injection of the sclerotic, nor extra lachrymation. On the third, (sometimes second) day the outermost layer of the cornea was shed in flakes, leaving ragged remnants behind which were finally thrown off, and the cornea appeared as smooth and clear as before. The opacity was at its most, fully as great as in your young specimen. I remember that I watched through an afternoon and half-a-night for the beginning of the process, and found the animal much disturbed and restless. If I remember aright it was this that led Mr. Kahn to examine and discover this appearance, and he observed it month after month with the same regularity, confirmed by myself.

I would add that the one I examined had no true *falx cerebri*, that there was a roundish irregular bony development apparently independent of the pia, over one of the trochlearis nerve roots, and that the pineal body in the hippopotamus is sharply pointed tongue-shaped and flat; a form not found by me in any other animal thus far.

Dr. Brill informs me that in removing the spinal cord of your hippopotamus he was struck by the coarseness and density of the spinal dura mater (*theca spinalis*). The arachnoid was filled in its meshes with a lymph-like gelatinous material, *i. e.* embryonic connective substance.''

The brain was given to Dr. W. R. Birdsall for examination. The period of gestation was seven months and twenty-three days.

The following measurements were taken :—

| | | | |
|---|---|---|---|
| Length from tip of nose to end of tail . . . . | 3 feet | 7½ | inches. |
| '' of head . . . . . . . . . . . . . . | | 10¼ | '' |
| '' '' body . . . . . . . . . . . . . . | 2 feet. | | |
| '' '' neck . . . . . . . . . . . . . . | | 4 | '' |
| '' '' tail . . . . . . . . . . . . . . . | | 5¼ | '' |
| Circumference at shoulders . . . . . . . . | 2 feet | 3 | '' |
| '' '' abdomen . . . . . . . . | 2 '' | 4½ | '' |
| Height at shoulder . . . . . . . . . . . . | 1 '' | 6¼ | '' |
| '' '' pelvis . . . . . . . . . . . . . . | 1 '' | 6½ | '' |
| Breadth of head between ears . . . . . . . | | 6 | '' |
| '' between eyes . . . . . . . . . . . | | 4¾ | '' |
| '' at nostrils . . . . . . . . . . . . | | 4 | '' |

Weight 65½ pounds.

